# The *Tomato Spotted Wilt Virus* Genome Is Processed Differentially in its Plant Host *Arachis hypogaea* and its Thrips Vector *Frankliniella fusca*

**DOI:** 10.3389/fpls.2016.01349

**Published:** 2016-09-07

**Authors:** Stephen J. Fletcher, Anita Shrestha, Jonathan R. Peters, Bernard J. Carroll, Rajagopalbabu Srinivasan, Hanu R. Pappu, Neena Mitter

**Affiliations:** ^1^Queensland Alliance for Agriculture and Food Innovation, The University of Queensland, St. LuciaQLD, Australia; ^2^School of Chemistry and Molecular Biosciences, The University of Queensland, St. LuciaQLD, Australia; ^3^Department of Entomology, College of Agricultural and Environmental Sciences, University of Georgia, TiftonGA, USA; ^4^Department of Plant Pathology, Washington State University, PullmanWA, USA

**Keywords:** tomato spotted wilt virus, *Arachis hypogea*, *Frankliniella fusca*, plant defense, plant–insect–virus interactions, vsiRNA, small RNA

## Abstract

Thrips-transmitted tospoviruses are economically important viruses affecting a wide range of field and horticultural crops worldwide. *Tomato spotted wilt virus* (TSWV) is the type member of the *Tospovirus* genus with a broad host range of more than 900 plant species. Interactions between these viruses and their plant hosts and insect vectors via RNAi pathways are likely a key determinant of pathogenicity. The current investigation, for the first time, compares biogenesis of small RNAs between the plant host and insect vector in the presence or absence of TSWV. Unique viral small interfering RNA (vsiRNA) profiles are evident for *Arachis hypogaea* (peanut) and *Frankliniella fusca* (thrips vector) following infection with TSWV. Differences between vsiRNA profiles for these plant and insect species, such as the relative abundance of 21 and 22 nt vsiRNAs and locations of alignment hotspots, reflect the diverse siRNA biosynthesis pathways of their respective kingdoms. The presence of unique vsiRNAs in *F. fusca* samples indicates that vsiRNA generation takes place within the thrips, and not solely through uptake via feeding on vsiRNAs produced in infected *A. hypogaea*. The study also shows key vsiRNA profile differences for TSWV among plant families, which are evident in the case of *A. hypogaea*, a legume, and members of Solanaceae (*S. lycopersicum* and *Nicotiana benthamiana*). Distinctively, overall small RNA (sRNA) biogenesis in *A. hypogaea* is markedly affected with an absence of the 24 nt sRNAs in TSWV-infected plants, possibly leading to wide-spread molecular and phenotypic perturbations specific to this species. These findings add significant information on the host–virus–vector interaction in terms of RNAi pathways and may lead to better crop and vector specific control strategies.

## Introduction

Thrips and tospoviruses continue to be one of the biggest production constraints to numerous crops in both developed and developing parts of the world, with an economic cost in excess of US$1 billion annually (Pappu et al., 2009). Tospoviruses have been reported to infect more than 1090 species in 85 families of monocots and dicots (Mandal, 2012). Several informative reviews on different aspects of tospoviruses, the only plant-infecting viruses in the family *Bunyaviridae*, and their thrips vectors have been published in recent years (Jones, 2005; Whitfield et al., 2005; Persley et al., 2006; Lebas and Ochoa-Corona, 2007; Tsompana and Moyer, 2008; Pappu et al., 2009; Riley et al., 2011). *Tomato spotted wilt virus* (TSWV) is a member of the *Tospovirus* genus and is ranked second among the top ten economically important viruses in the world (Scholthof et al., 2011). It is one of the most important pathogens affecting peanut production in the south-eastern United States (Culbreath and Srinivasan, 2011). At least 10 species of thrips (*Thysanoptera:Thripidae*) have been implicated in TSWV transmission in a persistent and propagative manner; that is, the virus also replicates in the insect vector (Ullman et al., 1993; Whitfield et al., 2005; Pappu et al., 2009). *Frankliniella fusca* Hinds (tobacco thrips) is considered to be an important vector of TSWV on peanut (Riley et al., 2011). Use of resistant cultivars along with the integration of cultural and chemical tactics including control of the insect vector through insecticides are the primary methods used to manage TSWV. Understanding the interactions between the host, vector and virus at a molecular level is a crucial component in designing new management strategies and remains a highly active area of research.

RNA interference (RNAi), a key conserved mechanism in eukaryotes, plays a major role in innate defense against viruses and transposons (Baulcombe, 2004; Pelaez and Sanchez, 2013). The trigger for RNAi is double-stranded RNA (dsRNA), which may be generated during viral replication in the host, be it plants or insects. DsRNA is processed into 21–24 nt small interfering RNAs (siRNAs) in a complex series of interactions involving Dicer-like proteins (DCLs), Argonauts (AGOs), and RNA-dependent RNA polymerases (RdRps) (Ding and Voinnet, 2007). Another form of RNAi produces 21–24 nt microRNAs (miRNAs) from imperfect hairpin structures known as pre-miRNAs. MiRNAs often target mRNA transcripts and thus have a role in gene regulation (Mallory and Vaucheret, 2004). Together, miRNAs and siRNAs molecules are classified as small RNAs (sRNAs).

Virus specific siRNAs (vsiRNAs) are generated via RNAi pathways in virus-infected plants, and have been identified in diverse eukaryotic species (Ding, 2010; Wu et al., 2010). The presence of abundant vsiRNA hotspots across the viral genome reveal the likely initiation and phased loci of the host’s RNAi targeting mechanisms. Previous reports have demonstrated that vsiRNA hotspots are not uniformly distributed across viral genome segments and gene-coding regions, suggesting that the host has a preference for targeting specific regions, with consequences for both viral pathogenicity and host specificity (Donaire et al., 2008; Wang et al., 2010; Mitter et al., 2013).

Due to replication of TSWV in the thrips vector, infection-induced changes in sRNA abundance along with the location and magnitude of vsiRNA hotspots relative to the plant host are also of interest. Reports of TSWV vsiRNA hotspot position and magnitude in insect vector-derived samples are largely absent from the literature, though studies of vsiRNA hotspots related to other viruses and insect hosts have been carried out (Myles et al., 2008; Mueller et al., 2010; Hess et al., 2011). Similar to the plant response to viral infection, inhibition of viral replication via the insect’s dsRNA-triggered RNAi machinery is well documented in *Drosophila melanogaster* (Meigen; Kemp et al., 2013), *Anopheles gambiae* (Giles; Keene et al., 2004) and *Aedes aegypti* (L.; Campbell et al., 2008). A comparative study of *Rice stripe virus*-infected plant hosts *Oryza sativa* (L.) and *Nicotiana benthamiana* (Domin) and insect vector *Laodelphax striatellus* (Fallen) demonstrated the similarities and differences in both overall sRNA abundance and vsiRNA-profiles in these plant and insect species (Xu et al., 2012). Differences in the biogenesis of sRNAs were readily evident, with 21 and 24 nt sRNAs most prevalent in the plant species, and 22 and 26–27 nt sRNAs in the insect vector. However, vsiRNAs aligning to the viral genome were dominated by the 21 nt sRNAs in the plant samples, and 22 nt sRNAs in the insect derived sample. Significant variation in the location and magnitude of vsiRNA hotspots between the hosts was readily evident, which may be more broadly indicative of virus–vector–host relationships.

The use of next-generation sequencing has allowed for comprehensive analysis of sRNA populations in virus-infected samples, both generally and the specific sRNA subset directed against the virus (Szittya et al., 2010; Xu et al., 2012; Miozzi et al., 2013; Mitter et al., 2013). In this study, deep sequencing of the small RNA component of samples derived from TSWV-infected and non-infected *Arachis hypogaea* (L.) host plants and the *F. fusca* (Hinds) vector was performed. *In silico* analysis of these data demonstrates both common and unique features among the plant host and insect vector, as well as between *A. hypogaea*, a legume, and members of the Solanaceae infected with the same viral isolate.

## Materials and Methods

### Maintenance of Non-infected and TSWV-Infected *A. hypogaea* Plants

*Arachis hypogaea* seedlings (cv. Georgia Green) were germinated and maintained in a growth room at 25–30°C, 40–50% relative humidity (rh) and L14:D10 photoperiod. Subsequently, sprouted seeds were transplanted and maintained in thrips-proof cages in a greenhouse at 25–30°C, 80–90% rh, and L14:D10 photoperiod. TSWV-infected volunteer *A. hypogaea* plants were collected from Belflower Farm (Coastal Plain Experimental Station, Tifton, GA, USA) and maintained in a greenhouse under the same environmental conditions. Leaflets with TSWV symptoms were collected, placed in a Munger cage, into which non-viruliferous *F. fusca* female adults were released (Munger, 1942). After the next generation adults emerged, potentially viruliferous adults obtained from the cages were used to inoculate prepared *A. hypogaea* plants. Ten female adults (up to 2 days old) were released on 1-week-old plants that had been dusted with 0.05 g of pine (*Pinus taeda* L.) pollen. Each plant was enclosed in a Mylar film (Grafix, Cleveland, PA, USA) cage with a copper mesh top. Plants were maintained in thrip-proof cages in the greenhouse as described above. Subsequently 3 weeks post thrips inoculation, infection status of the plants was determined by real-time quantitative reverse transcription polymerase chain reaction (qRT-PCR) using TSWV N-gene-specific forward and reverse primers, 5′-GCTTCCCACCC TTTGATTC-3′ and 5′-ATAGCCAAGACAACACTGATC-3′, respectively (Rotenberg et al., 2009). Plasmids with TSWV N-gene inserts were used as external copy number standards.

### Maintenance of Non-viruliferous and Viruliferous *F. fusca*

Adult *F. fusca* were initially collected from *A. hypogaea* blooms from Belflower farm. Subsequently, non-viruliferous and potentially viruliferous *F. fusca* colonies were separately reared on non-infected and TSWV-infected *A. hypogaea* leaflets, respectively, in Munger cages under the conditions described earlier. *F. fusca* reared for an entire generation (egg to adult) on TSWV-infected *A. hypogaea* leaflets alone were considered as potentially viruliferous. The TSWV infection status of subsamples from potentially viruliferous and non-viruliferous thrips used for this study was determined by qRT-PCR using the primers and conditions as described above. In both non-viruliferous and potentially viruliferous colonies, a majority of thrips (>95%) were females. Rotenberg et al. (2009) revealed that TSWV nucleocapsid RNA copies were two to three times greater in female thrips [*Frankliniella occidentalis* (Pergande)] when compared with male thrips. Although a different thrips species was the focus of this study [*F. fusca* (Hinds)], only females were used.

### Total RNA Extraction and Deep Sequencing

Approximately 160 female adults (up to 2 days old) were separately pooled from viruliferous and non-viruliferous thrips colonies, and total RNA was extracted. Similarly, leaf tissue from three TSWV-infected or non-infected plants (about 8 weeks old) was used for plant total RNA extraction. A total of two independent biological replicates were included for each *F. fusca* treatment and three independent biological replicates for each *A. hypogaea* treatment. Total RNA from thrips and plant samples were extracted using Direct-zol RNA Miniprep kit (Zymo Research, Irvine, CA, USA) using the manufacturer’s protocol. Library construction and sRNA sequencing (50 bp, single end) were carried out by the Beijing Genomic Institute using the Illumina HiSeq sequencing system. Samples were multiplexed and sequenced over two lanes. All sequence files are publicly accessible via the NCBI Sequence Read Archive (uninfected *A. hypogaea*: SRR3727343, SRR3727344, SRR3727345; TSWV-infected *A. hypogaea*: SRR3727346, SRR3727347, SRR3727348; uninfected *F. fusca*: SRR3727364, SRR3727365; TSWV-infected *F. fusca*: SRR3727366, SRR3727368).

### Bioinformatic Analysis of sRNA Sequence Files

Alignments of sRNA reads were carried out against the following TSWV RNA segment accessions: L RNA - NC_002052.1; M RNA - AY744483.1; S RNA - AY744475.1. For *A. hypogaea* sample alignments, reference sequences were obtained for *A. ipaensis*, a sequenced progenitor of *A. hypogaea*. These references included an annotated transcriptome (Araip.K30076.a1.M1) and mobile element data set (BB051914; Dash et al., 2016). Previously published raw sRNA sequence files from TSWV-infected *S. lycopersicum* (SRR947065) and *N. benthamiana* (SRR947064) were re-processed and used in this study for comparative purposes (Mitter et al., 2013). In this previous study, *S. lycopersicum* and *N. benthamiana* plants were mechanically inoculated with TSWV 3–4 weeks post-germination and leaf samples from TSWV positive plants were collected 17 days post inoculation.

Briefly, adapter sequences were removed from 50 bp single-end reads, which were then collapsed to identical sequences with maintained counts using the FASTX-Toolkit package. Only reads with lengths between 18 and 32 nt were retained for alignment normalization. Alignment of collapsed reads to reference sequences was performed using the SCRAM software package which allowed for exact matches to the reference sequence only (Fletcher, 2016). Normalization of aligned read count at each position was calculated as reads aligned per million reads between length 18 and 32 nt in the collapsed read file, allowing for robust quantitative comparison between alignments. Boxplots and sRNA length by count profiles were generated in R Studio using custom R scripts. Graphical representations of alignment profiles were produced either directly from the Python alignment software or via the Circos software package using tabulated output (Krzywinski et al., 2009). Scatter plots and Venn diagrams were generated in R Studio, and Euler diagrams in eulerAPE.

## Results And Discussion

**FIGURE 1:**
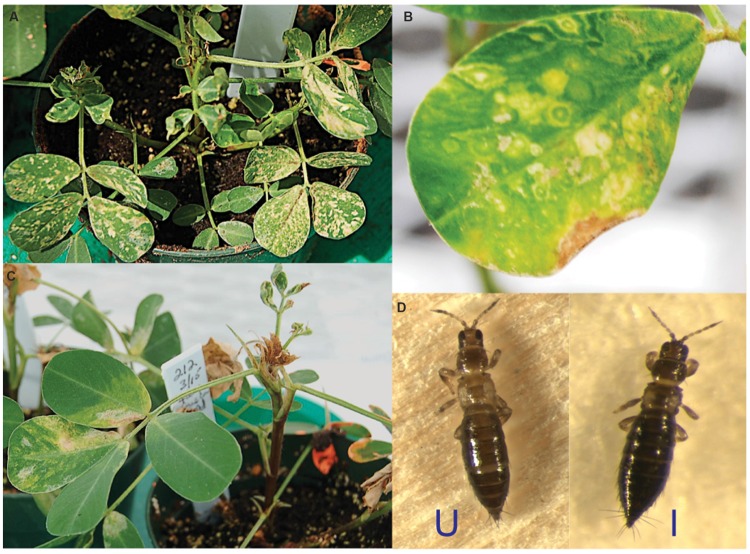
**Typical phenotype of *Tomato spotted wilt virus (*TSWV)-infected *Arachis hypogaea* and *Frankliniella fusca*. (A)** Chlorotic mottling on the *A. hypogaea* adaxial leaflet surface. **(B)** Concentric ringspots. **(C)** Drooping of leaf petioles and drying of terminals. **(D)** TSWV-infected *F. fusca* (I) displays no apparent virus-induced symptoms compared to its uninfected counterpart (U).

TSWV infection induced a wide range of symptoms in *A. hypogaea* plants in the greenhouse. Typical symptoms included chlorotic mottling on the adaxial leaflet surface (**Figure 1A**), concentric ringspots with green centers (**Figure 1B**), drooping of leaf petioles, and drying of terminals (**Figure 1C**). In contrast, viruliferous *F. fusca* displayed no obvious symptoms, with their external appearance indistinguishable from that of non-viruliferous *F. fusca* (**Figure 1D**).

### sRNA Profiles in *A. hypogaea* and *F. fusca* as a Result of TSWV Infection

**FIGURE 2:**
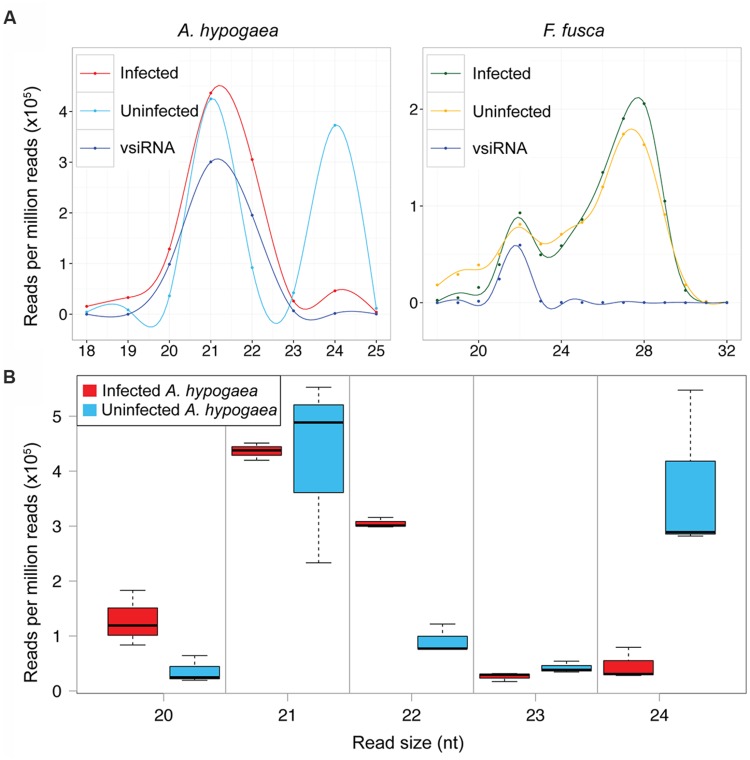
**Size distribution of TSWV-infected and uninfected *A. hypogaea* and *F. fusca* total sRNA and vsiRNA reads. (A)** Smoothed mean sRNA abundance profiles of TSWV-infected and uninfected *A. hypogaea* (left, *n* = 3 per treatment) and TSWV-infected and uninfected *F. fusca* (right, *n* = 2 per treatment). A disparate profile is evident for the infected versus uninfected *A. hypogaea* samples, with an almost complete loss of 24 nt small RNAs (sRNAs) in the infected plants, whilst little difference is apparent for the *F. fusca* samples across all sRNA sizes. vsiRNA profiles (not to scale) have a similar relative abundance of the 21–24 nt sizes to their infected host counterparts. **(B)** A boxplot of the total sRNA size distributions for TSWV-infected and uninfected *A. hypogaea* samples provides further detail, where an increase in 20 and 22 nt sRNAs along with a large decrease in the 24 nt sRNAs is evident in infected samples. Differences in abundance of 21 and 23 nt sRNAs are not apparent between infected and uninfected samples

The size distributions of sRNAs in TSWV-infected and uninfected samples are shown in **Figure 2A**. The uninfected samples represent only the host sRNAs whereas the infected samples include total sRNAs (host and vsiRNAs). We have also aligned only vsiRNAs in the infected samples. The categorisation of 20–24 nt total sRNAs into host sRNAs (non-vsiRNAs) and vsiRNAs is presented in Supplementary Table S1. The bimodal size distributions of sRNA reads in healthy *A. hypogaea* and *F. fusca* are typical of their origin, with 21 and 24 nt sRNAs dominating in the plant host and 22 nt and 27–28 nt sRNAs most prevalent in the insect vector (**Figure 2A**). The relative abundance of 21 and 24 nt peaks apparent in the healthy *A. hypogaea* profile closely resembled that of *N. benthamiana*, a Solanaceae member, in a previous report (Mitter et al., 2013). Similarly, the distribution of sRNA reads in the infected and healthy *F. fusca* samples matched those in profiles of insect species as diverse as the silkworm *Bombyx mori* (L.; Jagadeeswaran et al., 2010) and migratory locust *Locusta migratoria* (Wei et al., 2009). *L. striatellus* infected with *Rice stripe virus* also displayed a bipartite profile, though a peak was evident at 26–27 nt rather than 27–28 nt, and contrastingly, the abundance of the sRNAs making up the larger sized peak was lower than that of the 22 nt sRNAs (Xu et al., 2012). The distinct profiles of the plant host and insect vector likely reflect the differences in sRNA biogenesis and function among the organisms. The use of female thrips may have influenced the prevalence of vsiRNAs observed, as they could be more abundant in female thrips than in male thrips (Rotenberg et al., 2009).

Remarkably, the impact of TSWV infection on the size distribution of total sRNAs in *A. hypogaea* is profound, with a significant reduction in total 24 nt sRNAs after infection (**Figures 2A,B**). Compared to uninfected plants, there is also an increase in 22 nt sRNAs, and to a lesser extent, 20 nt sRNAs (**Figure 2B**). Such a change in relative abundance of 22and 24 nt sRNAs due to TSWV infection is in contrast to an increase in 24 nt sRNAs and decrease in 22 nt sRNAs in other reported species, such as *Bamboo mosaic virus*-inoculated *N. benthamiana* plants (Lin et al., 2010). A slight decrease in 24 nt sRNAs in cotton plants infected with *Cotton leaf roll dwarf virus* indicates a similar pattern, but at a much reduced magnitude compared to TSWV-infected *A. hypogaea* (Silva et al., 2011). These data suggest that infection by TSWV significantly alters the productive balance of the plant’s sRNA biogenesis machinery. In contrast, infection of the *F. fusca* vector by TSWV results in minimal change in the relative abundance of the various sRNA discrete sizes (**Figure 2A**).

### Alterations in the Endogenous sRNA Landscape of *A. hypogaea* Due to TSWV Infection

Due to their role in maintenance of genome stability via repression of transposable elements, the reduction in the number of 24 nt sRNAs in TSWV-infected *A. hypogaea* could have significant physiological consequences. Additionally, an increase in 22 nt sRNAs may lead to other further changes in endogenous sRNA abundance, as these sRNAs trigger biogenesis of phased sRNAs spanning complementary RNA sequences, along with silencing of the affected genes (McHale et al., 2013). Widespread perturbation of gene expression in infected plants, either directly or indirectly, may be a fundamental factor in the infected plant phenotype.

**FIGURE 3:**
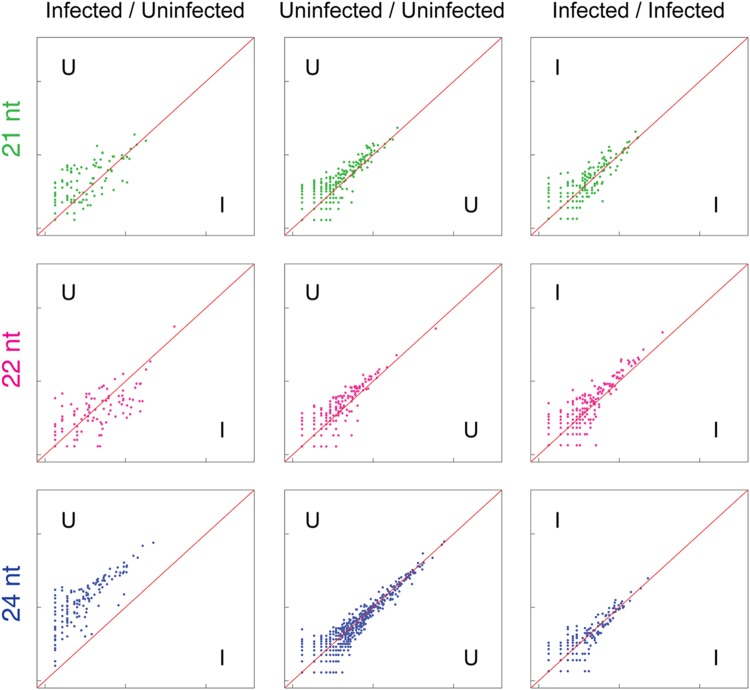
**Reduction of 24 nt sRNAs (blue) associated with repetitive elements in TSWV-infected *A. hypogaea* leaves.** Normalized sRNA alignment abundances for each discrete repetitive element are represented as *x* and *y* coordinates in each log-scale scatter plot. For uninfected vs. infected (U/I) plots, the alignment counts are the mean of two biological replicates for each treatment. For U/U and I/I plots, alignment counts are for the constituent single replicates of each treatment, with tight clustering around the diagonal an indication of low variation among biological replicates. The U/I plot for 24 nt sRNAs (bottom left) demonstrates a genome-wide reduction in 24 nt sRNAs complementary to repetitive elements in TSWV-infected plants. Increased scattering of points in the 21 and 22 nt U/I plots also indicates general variation in 21 and 22 sRNAs generated against repetitive elements in response to TSWV infection.

Analysis of sRNA alignments to repetitive and transposable elements within the *A. hypogaea* genome demonstrated a clear reduction in sRNAs derived from these elements in TSWV-infected plants (**Figure 3**). This shift likely has wide-ranging impacts, with transposition events potentially happening at a greater frequency, and the expression of nearby endogenous genes altered. In support of this prediction, previous studies have shown that the disruption of biogenesis of 24 nt sRNAs in plants had an extensive impact on the degree of DNA methylation along with the activity of targeted transposable elements, and the expression of neighboring genes (Jia et al., 2009; Groszmann et al., 2011).

**FIGURE 4:**
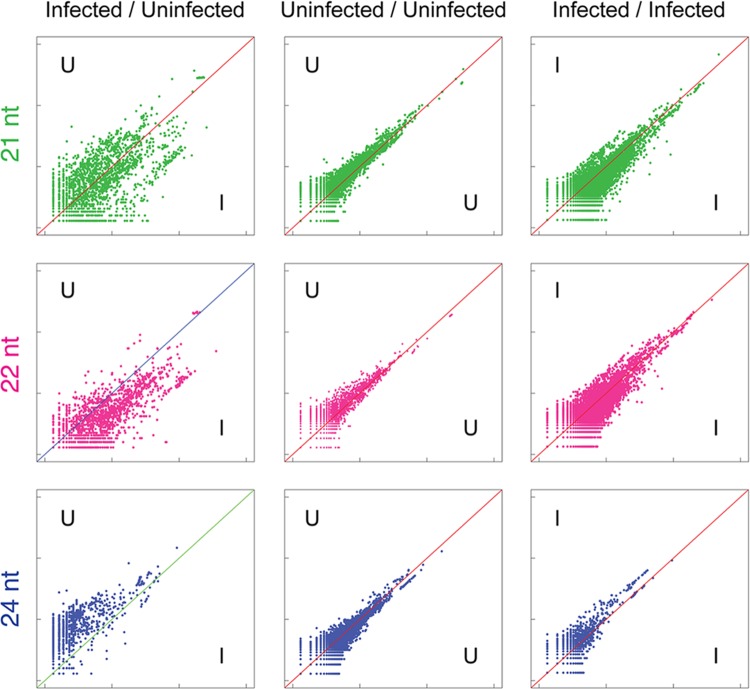
**Widespread variation in sRNA alignment abundance to cDNAs in uninfected versus TSWV-infected *A. hypogaea* leaves.** Normalized sRNA alignment abundances for each cDNA are represented as *x* and *y* coordinates in each log-scale scatter plot. For uninfected vs. infected (U/I) plots, the alignment counts are the mean of two biological replicates for each treatment. For U/U and I/I plots, alignment counts are for the constituent single replicates of each treatment, with tight clustering around the diagonal an indication of low variation among biological replicates. Similar to repetitive elements, a general reduction in 24 nt sRNAs aligning to cDNAs is evident in infected plants, though at a reduced level (bottom left). Importantly, the observed increased divergence of alignment counts in the 21, 22,and 24 nt U/I plots indicates widespread variation in sRNAs directed against gene sequences in uninfected versus infected plants.

Alignments to protein-coding sequences also demonstrated clear changes in the abundance of 21, 22, and 24 nt sRNAs targeting these sequences (**Figure 4**). Interestingly, an increase in the variance of coverage of coding sequences by 21, 22, and 24 nt sRNAs is particularly evident, pointing toward possible broad up- and down-regulation of gene expression in infected leaf tissue. A previous study has suggested that the TSWV-derived Non-structural protein (NSs) binds directly to intermediates of sRNA processing and potentially suppresses loading of sRNA into AGO complexes required for degradation or translational inhibition of complementary RNAs (Schnettler et al., 2010). Therefore, the disruption of sRNA levels observed in this study and the potential for NSs action on plant-generated sRNAs may be a means for perturbation of target RNA transcript levels and consequential physiological and developmental changes.

This report adds further evidence for the species-specific impact virus inoculation has on the dynamics of sRNA processing. The exact mechanism driving this shift in sRNA abundance and the reason it differs between species remains to be fully elucidated. DCL2, DCL3, and DCL4 homologs of plants all cleave dsRNA substrates, either endogenous or exogenous/viral derived dsRNAs, into 22, 24, and 21 nt sRNAs, respectively (Henderson et al., 2006; Bologna and Voinnet, 2014). Studies on *Arabidopsis dcl* mutants show DCL4 and DCL2 are partly redundant and act hierarchically in processing dsRNA substrates associated with post-transcriptional gene silencing (Parent et al., 2015). In contrast, DCL3 is accepted as the primary enzyme for generation of 24 nt siRNAs from dsRNAs produced by the Pol IV-RDR2 complex and associated with transcriptional silencing of repetitive elements and transposons (Gasciolli et al., 2005; Pontes et al., 2006; Wierzbicki et al., 2009; Johnson et al., 2014). Our data demonstrated that TSWV infection in *A. hypogaea*, but not the two Solanaceae species (Mitter et al., 2013), triggered a genome-wide depletion in 24 nt siRNAs associated with repetitive elements and transposons (**Figure 3**). This suggests that in *A. hypogaea* there was a TSWV-dependent suppression in expression levels, subcellular localization and/or the enzyme function of DCL3, or another upstream component of the transcriptional gene silencing pathway, such as Pol IV or RDR2 (Wierzbicki et al., 2009). Thus, inhibition of DCL3 or another component of the transcriptional gene silencing pathway is the most likely explanation for the shift in production of 24 nt siRNAs to 22 nt siRNAs in TSWV-infected *A. hypogaea*. Such a reaction to TSWV infection on the accumulation of 24 nt siRNAs clearly varies between plant species. Further expression analysis on components of the transcriptional gene silencing pathway may shed light on differential effects on TSWV infection in different host species.

### Divergence in Relative Abundance of *A. hypogaea* and *F. fusca* vsiRNAs Aligned to Each TSWV Genome Segment

The portion of total sRNAs that align to the infecting viral genome has been shown to vary greatly depending on the virus and the plant host (Dehaan et al., 1991; Bolduc et al., 2010; Mitter et al., 2013; Hong et al., 2015). Approximately 16% of all reads in each infected *A. hypogaea* replicate aligned to the TSWV genome, while just 0.4% aligned to the virus in the infected *F. fusca* vector (**Table 1**). The abundance of vsiRNAs aligning to the TSWV genome in *A. hypogaea* is high compared to *S. lycopersicum* and *N. benthamiana* infected with the same viral isolate (6% and 0.02%, respectively), though in the range of other host–virus combinations such as peach infected with *Peach latent mosaic viroid* (11.2 %; Bolduc et al., 2010) and rice infected with *Rice dwarf phytoreovirus* (∼22%; Hong et al., 2015).

**Table 1:** Effect of the host organism on *Tomato spotted wilt virus* (TSWV)-specific vsiRNA (viral small interfering RNA) levels.

Sample	Genome Size (Mb/1C)	Total Reads (average if multiple replicates)	vsiRNA reads per million reads (21–22 nt)	vsiRNA percentage of total (%)
*A. hypogaea*	∼2800	9179622	165298	16.53%
*F. fusca*	∼400	21915376	4197	0.42%
*S. lycopersicum*	∼950	19908563	62952	6.30%
*N. benthamiana*	∼3000	18780917	145	0.01%


*The mean percentage of total sRNA reads corresponding to the TSWV genome for infected Arachis hypogaea and Frankliniella fusca samples is displayed. S. lycopersicum and Nicotiana benthamiana alignment percentages are shown for comparison. All four species were infected with the same isolate of TSWV. The vsiRNA load in the A. hypogaea host is ∼30 times that of the F. fusca vector.*

Tospoviruses are characterized by a tripartite genome structure consisting of large (L), medium (M) and small (S) single-stranded RNA segments with sense and ambi-sense coding frames. The L segment consists of a large open reading frame (ORF) that encodes *RdRp* (RNA-dependent RNA polymerase), which is required for viral replication (Dehaan et al., 1991). ORFs for *Gn/Gc* (precursor for the *N*-terminal G2 and *C*-terminal G1proteins) and *NSm* (non-structural protein) occur in the M segment. NSm is required for cell-to-cell viral movement (Soellick et al., 2000), and Gn/Gc for synthesis of glycoproteins present in the viruses’ lipid envelope (Snippe et al., 2007). The S segment encodes the *N* (nucleocapsid) gene, which encapsidates the RNA segments (Dehaan et al., 1990), and *NSs* (non-structural/suppressor of silencing protein), which is of particular importance in the defense against the plant’s RNAi defense strategy. Indeed, the degree of NSs protein accumulation in plant tissues has been associated with the severity of TSWV infections (Takeda et al., 2002). NSs can bind to both dsRNA and vsiRNA targets, allowing it to block antiviral RNA silencing before and after DCL-mediated dsRNA cleavage (Schnettler et al., 2010). Importantly, NSs has been shown to be effective in not only plant hosts, but also in insect vectors (Margaria et al., 2014), possibly through its affinity for longer dsRNA forms. Alignment of reads from infected plant samples to individual genome segments of TSWV has been previously reported, also with dissimilar results depending on the plant species and viral isolate (Mitter et al., 2013; Margaria et al., 2015).

**FIGURE 5:**
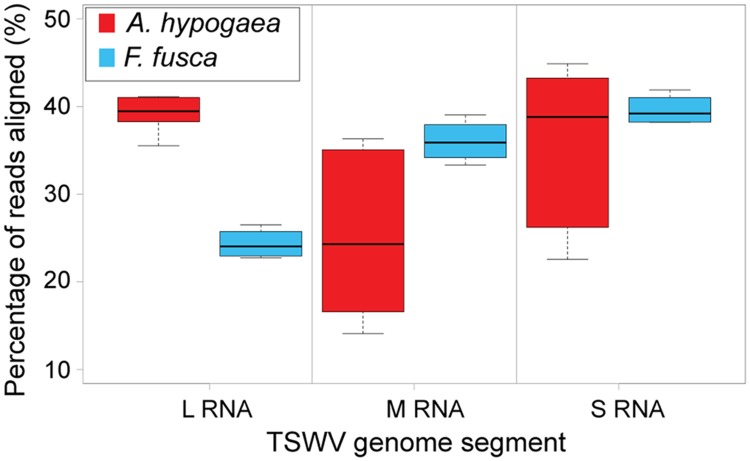
**Boxplot of the percentage of total 21 and 22 nt vsiRNAs aligned to each TSWV RNA segment [*A. hypogaea n* = 6 (3 samples^∗^2 vsiRNA sizes); *F. fusca n* = 4 (2 samples^∗^2 vsiRNA sizes)].** Unlike the large segment, significant variation is evident in the alignments to the medium and small segments amongst TSWV-infected *A. hypogaea* samples, indicating no clear vsiRNA preference for either segment.

In this study, TSWV-aligned vsiRNAs from *A. hypogaea* demonstrated a distribution distinct from vsiRNAs from the Solanaceae hosts, with a generally high portion aligned to the L segment and variable percentages aligning to the M and S segments, depending on the replicate. In contrast, *F. fusca*-derived vsiRNAs more closely matched the aligned vsiRNA distribution from *S. lycopersicum* and *N. benthamiana* (**Figure 5**; **Table 2**).

**Table 2:** Mean percentage of aligned reads for each TSWV segment along with the expected alignment percentage based on the size of the segment.

Genome segment	Expected percentage alignment based on size	Mean percentage of 21 – 22 nt vsiRNAs aligned – *A. hypogaea*	Mean percentage of 21 – 22 nt vsiRNAs aligned – *F. fusca*
L RNA	54%	39%	24%
M RNA	29%	25%	36%
S RNA	18%	36%	40%


*The abundance of aligned vsiRNAs in both A. hypogaea and F. fusca samples diverge from these expected percentages, demonstrating a segment-specific targeting preference.*

**FIGURE 6:**
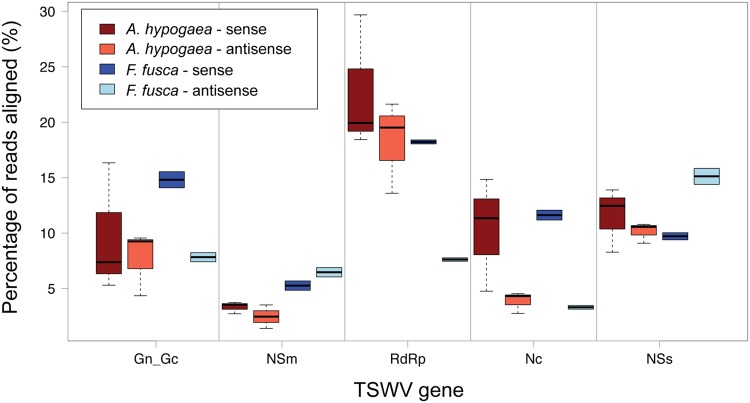
**Boxplot of sense and anti-sense read alignments for each TSWV gene.** No clear preference for polarity is evident for TSWV-infected *A. hypogaea*. Individual genes do display a preference in TSWV-infected *F. fusca*, with a sense preference for *GnGc*, *RdRp* and *Nc*, and an antisense preference for *NSm* and *NSs*.

The use of multiple fully independent biological replicates for *A. hypogaea* sRNA sequencing provides a detailed picture of the strand polarity of alignments to individual TSWV gene sequences (**Figure 6**). Significant variation amongst the *A. hypogaea* replicates indicates that there is little preference for a particular alignment orientation. This is in contrast to earlier reports for TSWV, where a greater abundance of vsiRNAs generally aligned to the sense strand of the virus in Solanaceae hosts (Mitter et al., 2013). The possibility exists that greater replication of sequencing for other TSWV-infected plant species may demonstrate similar variation. *F. fusca*, on the other hand, shows a sense preference for the *Gn/Gc*, *RdRp* and *Nc* genes, and an antisense preference for the *NSm* and *NSs* genes.

Due to vsiRNA being processed from dsRNA intermediates, it could be expected that vsiRNAs are equally distributed toward sense and antisense strands. The observed strand bias of vsiRNAs in *F. fusca* may be due to vsiRNAs binding to highly abundant complimentary viral transcripts, as has been suggested in previous studies (Smith et al., 2010; Harris et al., 2015). Such binding could potentially sequester and remove vsiRNA from the pool for sRNAs submitted for sequencing. If such an explanation were correct, it is predicted that the difference between strand biases observed for *A. hypogea* compared to *F. fusca* may be due to host-specific differences in the level of viral RNA transcript expression and/or availability.

### vsiRNA Preference and Hotspot Locations Evident in Alignment Profiles

**FIGURE 7:**
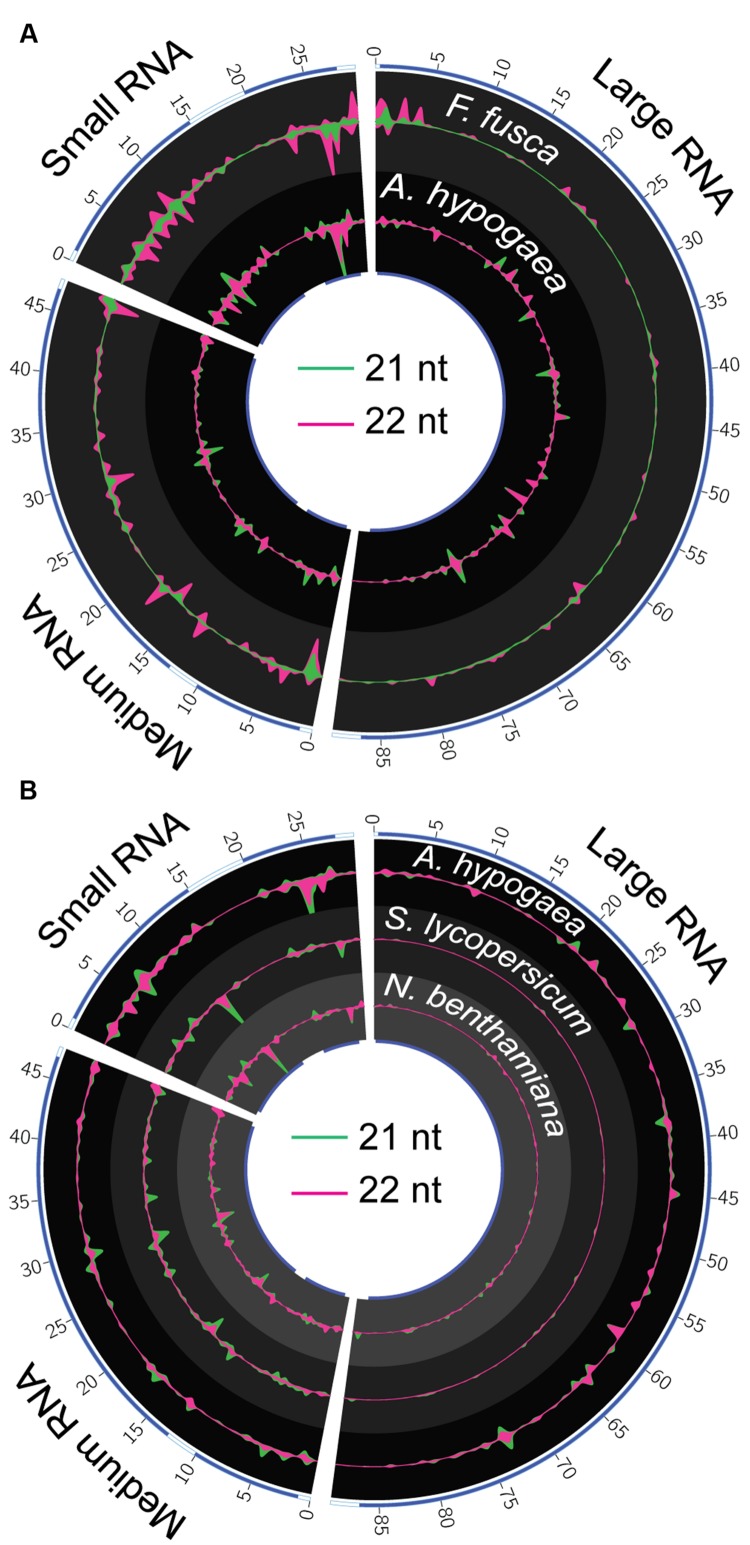
**Circos plots of 21 and 22 nt TSWV-associated vsiRNA hotspots in *A. hypogaea*, *F. fusca, S. lycopersicum and N. benthamiana*. (A)** 22 nt vsiRNA (violet) hotspots dominate in the *F. fusca* track (scale = ±2.5 RPMR), while 21 nt (green) vsiRNAs dominate in the *A. hypogaea* track (scale = ±55 RPMR). Blue bands on the inner and outer tracks represent the positions of gene-coding regions. The magnitude of vsiRNA hotspots is greatest in the sRNA segment in the *F. fusca* profile. Hotspots are more evenly spread across all segments in the *A. hypogaea* profile. **(B)** A comparison of *A. hypogaea* hotpots (scale = ±55 RPMR) again shows a more evenly spread distribution compared to TSWV-infected *S. lycopersicum* (scale = ±50 RPMR) and *N.benthamiana* (scale = ±0.1 RPMR) samples. vsiRNA hotpots are virtually absent in the large segment of the *S. lycopersicum* and *N. benthamiana*, but readily apparent in *A. hypogaea*, possibly indicating targeting of RdRP in this species.

The location and magnitude of vsiRNA hotspots spanning the TSWV genome in *A. hypogaea* and *F. fusca* samples are partially similar, though key differences exist (**Figure 7A**). In general, 21 and 22 nt vsiRNA hotspots within each species are co-located, with 21 nt peaks of greater magnitude in *A. hypogaea*, and 22 nt peaks larger in *F. fusca*. Visualization of vsiRNA coverage via circos plots demonstrates that relative to *F. fusca*, hotspots are more evenly dispersed across each genome segment in *A. hypogaea*. The *F. fusca* plots show fewer easily discernible peaks in the large RNA segment, and a greater abundance in the sRNA segment. Hotspots often occur in coding regions of the genome for both species, with a distinct gap apparent between adjacent genes located on the sRNA segment. Whilst hotspots do sometimes occur in the same locus in both the plant host and insect vector, many are unique to each organism; reinforcing that RNAi targeting mechanisms do have species-specific preferences. Importantly, these differences also indicate that TSWV-specific sRNAs in the insect are not simply derived from the plant itself via feeding, and are unique to the insect vector.

A comparison of *A. hypogaea* vsiRNA hotspots with those previously reported for *S. lycopersicum* and *N. benthamiana* infected with the same TSWV isolate (Mitter et al., 2013) shows distinct differences between plant families (**Figure 7B**). Similar to *F. fusca*, the vsiRNA hotspots in the L RNA segments corresponding to the *RdRP* gene are lacking in *S. lycopersicum* and *N. benthamiana*, indicating a very specific focus for *A. hypogaea*. Accordingly, though the external symptoms of TSWV infection are comparable in severity between *A. hypogaea* and *S. lycopersicum*, this *A. hypogaea*-specific direction of vsiRNAs toward the TSWV’s *RdRP* sequence may have significant downstream impacts upon pathogenicity relative to the Solanaceae.

The biogenesis and specific effects of vsiRNAs that form hotspots in alignment profiles are an area of ongoing study. Highly expressed vsiRNAs are thought to be a result of advantageous structural conditions in the confined regions of dsRNAs that vsiRNAs are generated from (Zhang et al., 2015). Differences in vsiRNA hotspot prevalence among plant families infected with the same viral isolate indicate that the localized environment the virus inhabits varies enough to impact RNA secondary structure, and thus the relative makeup of vsiRNAs generated.

**FIGURE 8:**
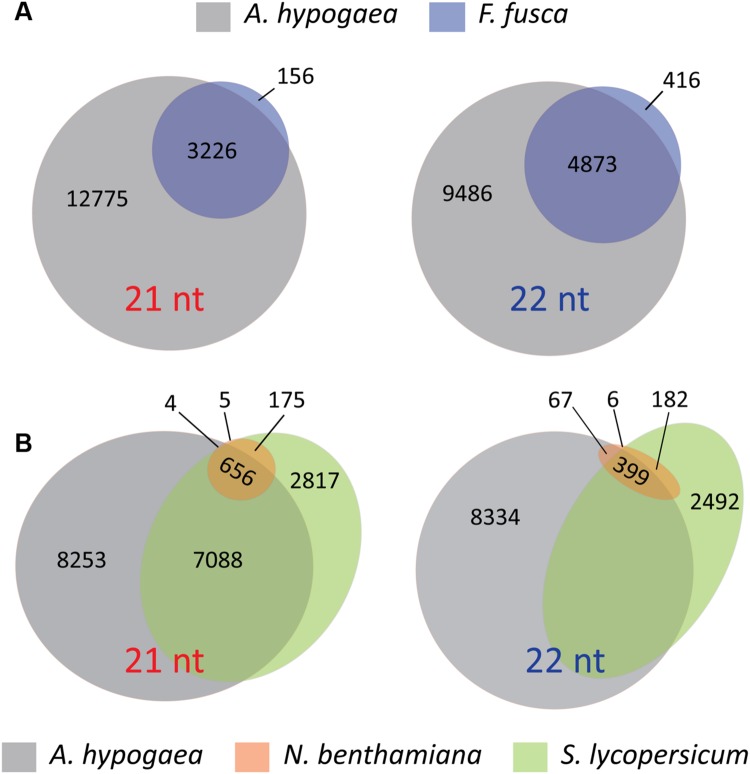
**Venn and Euler diagrams signifying the number of discrete 21t and 22 nt vsiRNAs from all read files that are common and unique to each species. (A)** Venn diagrams of 21 and 22 nt vsiRNAs common and unique to *A. hypogaea* and *F. fusca*. Whilst many vsiRNAs are common, the presence of unique vsiRNAs in the *F. fusca* samples indicates that the sampled vsiRNAs are at least partly generated by the thrips, and not solely via the uptake from TSWV-infected plant material. **(B)** Euler diagrams of 21 and 22 nt vsiRNAs common and unique to *A. hypogaea*, *N. benthamiana* and *S. lycopersicum*. The differences in sRNA biogenesis between the *A. hypogaea* and the Solanaceae are evident, with a high portion of unique reads for each. These data further support a divergent RNAi-mediated response to TSWV infection for each family.

The presence and absence of discrete vsiRNAs that are unique or shared between host and vector, or between different plant species infected with TSWV, reinforce the interpretations of the hotspot analyses. Unique vsiRNAs are readily apparent in *F. fusca* samples, further supporting the indication that vsiRNA generation takes place within the thrips, and not solely through uptake via feeding on vsiRNAs produced in infected *A. hypogaea* leaf tissue (**Figure 8A**). Additionally, the differences between *A. hypogaea* and the Solanaceae remain pronounced (**Figure 8B**). Due to the low TSWV vsiRNA load in *N. benthamiana*, almost all vsiRNAs present are common to *S. lycopersicum*. However, a similar number of discrete vsiRNAs only show partial overlap between *A. hypogaea* and *N. benthamiana*/*S. lycopersicum*, reinforcing the idea of a distinctive RNAi-mediated response to TSWV infection for different host plant families.

## Conclusion

The unique sRNA and TSWV-induced vsiRNA profiles of the host plant *A. hypogaea* and thrips vector *F. fusca* are of specific interest in understanding the mechanisms of viral infection and repression. The relative abundance of sRNAs in the non-infected plant and insect samples, along with the 21 and 22 nt vsiRNAs in TSWV-infected samples, reflect the different means of sRNA biosynthesis in these kingdoms. Phenotypic changes resulting from TSWV infection in the thrips vector are not readily apparent, and indeed the abundance of generated vsiRNAs is relatively low. In contrast, infection with the same viral isolate results in a large-scale changes in the peanut host, from overall reductions of 24 nt sRNAs and increases in 22 nt sRNAs, to up- and down-regulation of sRNAs directed against repetitive elements and endogenous genes. The mechanism behind these perturbations, along with their full impact on gene expression and genome stability, are yet to be elucidated, but indicate that *A. hypogaea* is a species of particular significance in virus/host interaction studies.

## Author Contributions

SF, AS, BC, RS, HP, and NM conceived and designed experiments. AS and SF performed experiments. SF, AS, and JP analyzed the data. All authors contributed to the writing, discussion, and approval of the final manuscript. SF and AS are to be considered as equal joint first authors.

## Funding

This work was supported by the National Peanut Board (grant: NPB-2521RF330498) at the University of Georgia, the College of Agricultural, Human and Natural Resource Sciences, Agricultural Research Center at Washington State University (project WNPO 0545), and by Horticulture Innovation Australia Limited at the University of Queensland.

## Conflict of Interest Statement

The authors declare that the research was conducted in the absence of any commercial or financial relationships that could be construed as a potential conflict of interest.
