# Supplementary material for: The Tomato Spotted Wilt Virus Genome Is Processed Differentially in its Plant Host Arachis hypogaea and its Thrips Vector Frankliniella fusca
Source: Front Plant Sci. 2016 Sep 7;7:1349. doi: 10.3389/fpls.2016.01349 (PMC5013717; doi:10.3389/fpls.2016.01349)
Supplement: Supplementary file 1 [file Table_1.DOCX]

**Supplementary Table S1: Average 21 to 24 nt read counts for host sRNAs (non-vsiRNAs) and vsiRNAs present in TSWV-infected and uninfected *A. hypogaea* and *F. fusca* samples.** Average sRNA abundance is normalised as reads per million reads (RPMR). vsiRNAs are absent in uninfected samples. Differences in abundance of host sRNAs between infected and uninfected samples are not solely accounted for by the presence or absence of vsiRNAs.

| **sRNA length** | **Infected *A. hypogaea*** | | **Uninfected *A. hypogaea*** | | **Infected *F. fusca*** | | **Uninfected *F. fusca*** | |
| --- | --- | --- | --- | --- | --- | --- | --- | --- |
|  | **vsiRNA (average RPMR)** | **Host sRNA (non vsiRNA) (average RPMR)** | **vsiRNA (average RPMR)** | **Host sRNA (non vsiRNA) (average RPMR)** | **vsiRNA (average RPMR)** | **Host sRNA (non vsiRNA) (average RPMR)** | **vsiRNA (average RPMR)** | **Host sRNA (non vsiRNA) (average RPMR)** |
| **20** | 32855 | 95722 | 0 | 36200 | 76 | 15715 | 0 | 39016 |
| **21** | 100172 | 336179 | 0 | 424830 | 1222 | 38101 | 0 | 50131 |
| **22** | 65126 | 240078 | 0 | 91968 | 2975 | 89876 | 0 | 81000 |
| **23** | 2249 | 23732 | 0 | 42282 | 82 | 49406 | 0 | 60589 |
| **24** | 538 | 45575 | 0 | 372809 | 15 | 58829 | 0 | 70678 |
